# The Spectrum of Malnutrition/Cachexia/Sarcopenia in Oncology According to Different Cancer Types and Settings: A Narrative Review

**DOI:** 10.3390/nu13061980

**Published:** 2021-06-09

**Authors:** Paolo Bossi, Paolo Delrio, Annalisa Mascheroni, Michela Zanetti

**Affiliations:** 1 Medical Oncology Unit, ASST Spedali Civili di Brescia, 25123 Brescia, Italy; 2 Department of Medical and Surgical Specialties, Radiological Sciences, and Public Health, University of Brescia, 25123 Brescia, Italy; 3 Colorectal Surgical Oncology, Istituto Nazionale per lo Studio e la Cura dei Tumori, Fondazione Giovanni Pascale IRCCS-Italia, 80131 Naples, Italy; delrio.paolo@gmail.com; 4 Clinical Nutrition and Dietetics Unit, ASST Melegnano-Martesana, 20077 Melegnano, Italy; annalisa.mascheroni@asst-melegnano-martesana.it; 5 Department of Medical Sciences, University of Trieste, 34100 Trieste, Italy; zanetti@units.it

**Keywords:** malnutrition, cachexia, sarcopenia, treatment type, stage, cancer subtype

## Abstract

Nutritional status in oncological patients may differ according to several modifiable and non-modifiable factors. Knowledge of the epidemiology of malnutrition/cachexia/sarcopenia may help to manage these complications early in the course of treatment, potentially impacting patient quality of life, treatment intensity, and disease outcome. Therefore, this narrative review aimed to critically evaluate the current evidence on the combined impact of tumor- and treatment-related factors on nutritional status and to draw some practical conclusions to support the multidisciplinary management of malnutrition in cancer patients. A comprehensive literature search was performed from January 2010 to December 2020 using different combinations of pertinent keywords and a critical evaluation of retrieved literature papers was conducted. The results show that the prevalence of weight loss and associated symptoms is quite heterogeneous and needs to be assessed with recognized criteria, thus allowing a clear classification and standardization of therapeutic interventions. There is a large range of variability influenced by age and social factors, comorbidities, and setting of cures (community-dwelling versus hospitalized patients). Tumor subsite is one of the major determinants of malnutrition, with pancreatic, esophageal, and other gastroenteric cancers, head and neck, and lung cancers having the highest prevalence. The advanced stage is also linked to a higher risk of developing malnutrition, as an expression of the relationship between tumor burden, inflammatory status, reduced caloric intake, and malabsorption. Finally, treatment type influences the risk of nutritional issues, both for locoregional approaches (surgery and radiotherapy) and for systemic treatment. Interestingly, personalized approaches based on the selection of the most predictive malnutrition definitions for postoperative complications according to cancer type and knowledge of specific nutritional problems associated with some new agents may positively impact disease course. Sharing common knowledge between oncologists and nutritionists may help to better address and treat malnutrition in this population.

## 1. Introduction

Malnutrition is a frequent hallmark of cancer patients, resulting in unintentional weight loss due to a lack of intake or uptake of nutrients [1]. This condition has a definitive impact on several aspects of cancer treatment and outcome: reducing treatment intensity, increasing treatment toxicities, worsening patients’ quality of life, and ultimately jeopardizing their survival.

Patients’ nutritional issues should be considered as a continuum, in an ideal line from the first signs and symptoms of anorexia to precachexia, cachexia, and refractory cachexia. As in a pyramid, refractory cachexia represents the highest part, while the vast basis is made up of initial nutritional impairment [2]. However, it is well known that the efficacy and the impact of any nutritional interventions are linked to the timing of the support, with the best results obtained with the early intervention [3].

Therefore, it is essential to periodically assess cancer patients during the different phases of the treatment journey. As a matter of fact, nutritional status is not a fixed condition, but a changeable status. Tumor stage, treatment type and setting, and comorbidities influence the nutritional needs of the patient, requiring a continuous evaluation of the nutritional, inflammatory and metabolic pathways. In addition, there are huge differences among tumor types and treatment settings, so that one can tailor the nutritional interventions according to the existing risk.

Moreover, the definitions of malnutrition, cachexia and sarcopenia have not been uniformly employed within clinical reports, therefore often complicating the identification of their prevalence among cancer patients. Malnutrition is present with the diagnosis of cancer in about 15–40% of cases and this incidence increases during treatment, characterizing 40–80% of the patients in this phase [4]. Malnutrition increases the risk of toxicity, worsens quality of life, and decreases patients’ functionality. Moreover, it is strictly linked to sarcopenia in that it worsens muscle function, causing a decrease in lean body mass and muscle performance. Weight loss and loss of skeletal muscle mass are two hallmarks of cancer cachexia, a well-known, gradual, and irreversible process in advanced cancer patients.

In this complex scenario, this narrative review aimed to evaluate the current evidence on the combined impact of tumor- and treatment-related factors on nutritional status with a particular emphasis related to the subsite of disease and phase of treatment, in order to help depict the heterogeneous pattern of malnutrition and to provide some practical conclusions to support the multidisciplinary management of this condition. We performed a critical evaluation of the literature papers from January 2010 to December 2020, giving an overview of the complex pattern and relationships existing between malnutrition, sarcopenia and cachexia in cancer patients. A comprehensive literature search was conducted using MEDLINE, with different combinations of pertinent keywords. The following terms were considered: “malnutrition”, “sarcopenia”, “cachexia”, “cancer”, “surgery”, “radiation therapy”, “chemotherapy”, “targeted agents”, “immunotherapy”, and “palliative care”. Authors were asked to identify further references from their personal collection of literature or other sources and they chose the most relevant manuscripts to be included in the present review. Each author then drafted a section of the review, comprising the evidence from literature and a critical evaluation according to their personal clinical experience on the topic. Each contribution was then reviewed and harmonized with the others by all the authors and a final version of the paper was circulated and approved.

## 2. Tools to Identify Nutritional Issues and Sarcopenia

Early recognition of malnutrition is essential for the correct management of the cancer patient. To identify and treat patients with malnutrition or those at high nutritional risk, a nutritional screening and a full nutritional assessment should be performed [5].

Nutritional screening should be performed at the time of the diagnosis, preferably before starting anticancer treatments. Several validated screenings tools are available for identifying a malnutrition status or a risk of developing malnutrition, for instance (a) the Nutritional Risk Screening 2002 (NRS 2002), (b) the Malnutrition Universal Screening Tool (MUST), and (c) the Mini Nutritional Assessment (MNA). Nutritional screenings should be repeated regularly throughout the therapeutic process, especially in cancer types with a high impact on nutritional status [6].

Patients at risk of malnutrition, according to the results of nutritional screening, should be referred to a clinical nutrition service for nutritional assessment and treatment.

Nutritional assessment for malnutrition and sarcopenia should include:

- Anthropometric measurements: Actual body weight, height, body mass index (BMI).
- Weight loss evaluation: An unintentional weight loss of >5% in the last six months is considered clinically relevant [7].
- Assessment of body composition through bioelectrical impedance vector analysis (BIVA): As a matter of fact, it allows a more detailed understanding of hydration status and cell mass that may be modified by pathological conditions. Moreover, the determination of phase angle seems to be a predictive outcome parameter in cancer patients [8]. Lean mass determination can also be performed by dual-energy X-ray absorptiometry (DXA), a low-dose radiation technique that allows the direct measurement of the various body compartments [9].

Computed tomography (CT) and magnetic resonance imaging (MRI) constitute the gold standard techniques to assess body composition [10,11]. The tomography image of the third lumbar vertebra (L3) is the most used method to measure and provide an accurate estimate of skeletal muscle mass. Single abdominal slice (L3) on MRI has been demonstrated to correlate with total skeletal muscle and adipose tissue [12,13,14,15]. However, both techniques are not yet feasible on a large scale [16].

- Biochemical data related to inflammatory and metabolic status: serum albumin, prealbumin, total lymphocyte count cholesterol, C reactive protein (CRP), transferrin, interleukin-6 (IL-6), and fibrinogen.
- Evaluation of nutritional intake, appetite, resting energy expenditure (REE) using indirect calorimetry, physical activity levels using metabolic holters [17].
- Evaluation of sarcopenia parameters [18]: muscle strength using a handgrip dynamometer [19] and chair stand test; muscle quantity using BIVA [20], DEXA and CT [21,22]; physical performance measures using tests as gait speed, short physical performance battery, timed-up-and-go test (TUG), and 400-m walk [23].
- Quality of Life and functional skills through specific questionnaires.

The accuracy of nutritional status determination is achievable with the combination of the described parameters. In this regard, the use of simple anthropometric measures may not provide information on body composition alterations, especially on the reduction of muscle mass, which may occur regardless of weight loss or BMI in cancer patients [10,24]. Low muscle mass evaluation in overweight or obese cancer patient is still a challenging task [25].

In 2016, the GLIM criteria for malnutrition diagnosis were identified [26]. Firstly, it is necessary to identify a nutritional status “at risk” through one of the validated screening tools. Secondly, it is mandatory to perform an assessment aimed at the diagnosis and staging of the malnutrition condition. There are five main diagnostic criteria: three phenotypic criteria (unintentional weight loss, low body mass index, reduced muscle mass) and two etiological criteria (reduced food intake or absorption, inflammation or co-morbidities). Phenotypic criteria are summarized in Table 1, whilst etiologic criteria are represented in Table 2.

The diagnosis of malnutrition requires at least one phenotypic criterion and one etiologic criterion. Phenotypic criteria are also used to divide the severity of malnutrition into Stage 1 (moderate) or Stage 2 (severe), as shown in Table 3.

Novel approaches to the diagnosis of sarcopenia have been evaluated to determine muscle mass, muscle function, skeletal muscle index (SMI) and impact on Qol [12,27,28] These methods consist of CT-based alternative lumbar measurements [13,29,30,31], ultrasounds assessment [32,33], specific biomarkers [34,35], score-based approaches [36], and Qol questionnaires [37,38].

These tools need to be validated, reliable, and accurate for future use in clinical practice [39,40]. Global consensus on the definition and diagnostic criteria of sarcopenia are also necessary to allow wider use of these tests.

## 3. Magnitude of the Problem according to Cancer Types

Malnutrition may affect as many as 75% of cancer patients [41,42] with a wide range of prevalence. This large range of variability is influenced by cancer-related (type, stage and treatment), demographic (age) and social factors (community-dwelling versus hospitalized patients). It has already been established that, in relation to the same cancer type, studies assessing malnutrition in hospital settings may report higher prevalence as compared to those performed in the community setting due to disease severity and to the distinct contribution of hospital-related malnutrition [43]. Moreover, patients at advanced stages of disease generally display a higher prevalence of overt malnutrition as compared to those in earlier stages [2]. In addition, the screening tool adopted to diagnose malnutrition may influence the prevalence rate [44], as well as the adoption of criteria that include the assessment of body composition to detect low muscle mass, such as computed tomography, DEXA, or BIA.

However, despite this mixture of factors influencing the nutritional status and the diagnosis of malnutrition, specific cancer types have been consistently associated with a higher risk of malnutrition and of developing cachexia. This condition is the result of tumor-induced activation of inflammatory pathways [45], which triggers a wasting response characterized by anorexia, altered metabolism, and involuntary loss of lean and fat mass that finally result in cachexia [7,46,47,48,49,50]. The magnitude of the systemic inflammatory response and the risk of developing cachexia are linked to several factors but most important to tumor type [51]. Specific tumors, such as lung and pancreas present distinct gene expression profiles of cachexia-inducing factors that may explain why these cancer types are more prone to develop a wasting syndrome [52]. Cachexia is a strong prognostic marker of adverse clinical outcomes, as demonstrated by the observation that, at least in lung cancer, a weight loss ≥2% has been associated with poor overall and progression-free survival [53].

Malnutrition is also very common in cancers that affect gastrointestinal function (i.e., swallowing and digestive ability), such as esophagus and stomach neoplasms. In this group of tumors, however, the concomitant involvement of systemic inflammation in malnutrition and cachexia has been demonstrated [54].

Many studies addressing the overall prevalence of malnutrition according to cancer type in different countries and settings have been published over the years and are reported in Table 4. When interpreting the results of these studies, one hurdle is represented by the methodology used to define malnutrition, either scoring systems to identify increased nutritional risk or tools to directly assess malnutrition, since anthropometric, clinical, and laboratory variables have been used. Despite this limitation, most of these studies are concordant in confirming that the highest risk of malnutrition is carried by gastroesophageal, pancreas, and head and neck tumors.

It should be mentioned that these tumor types are associated with protein-energy malnutrition and cachexia. Malnutrition in the context of obesity is notably a risk factor for the development and recurrence of other types of cancers (such as gynecologic and colon tumors) [55] although some studies have challenged this concept suggesting that the so-called “obesity paradox”, i.e., increased survival at higher BMIs also applies to some cancer types. The paradox, however, can be simply explained by methodological, clinical, and statistical considerations and does not apply if alternate measures of body mass and composition are used [56]. The importance of body composition and sarcopenia rather than of crude BMI on clinical outcomes in cancer patients is particularly evident for sarcopenic obesity. This condition is often underdiagnosed and challenging as to its management, which should be prompt and aggressive in order to improve survival and to avoid complications of cancer therapy [11,57]. The mechanisms linking obesity, diet and hormones and tumor initiation and progression will not be discussed in this review. Similarly, epidemiological and clinical data regarding sarcopenic obesity and cancer will not be presented.

**Table 4:** Summary of studies assessing the prevalence of malnutrition in cancer (any type) according to the tumor site.

Study	Design	Country	Sample size	Age, years	Setting	Malnutrition Assessment	Cut off for Malnutrition	Malnutrition Prevalence (%)
Pressoir 2010 [58]	Prospective	France	1545	Mean 59.3 ± 13.8	Hospital and Outpatient Clinic	Nutricode and recommendation of the National Health Authority	Age ≤ 70 years: Weight loss (WL) in 6 months >10% or BMI < 18.5 Age > 70 years: WL in 6 months ≥10% or BMI < 21	Upper digestive: 49.5 Head and Neck: 45.6 Lung: 40.2 Hematology: 34.2 Gynecology: 32 Colorectal: 31.2 Others: 27 Breast: 18.3
Bozzetti 2012 [59]	Prospective	Italy	1453	Median 64.0 (55–71)	Outpatient	Nutritional Risk Screening (NRS 2002)	≥3	Oesophagus: 62.5 Pancreas: 54.3 Stomach: 43.7 Upper respiratory airways: 28.6 Oral cavity: 28.5 Lung: 28.1 Other: 25.2 Colon-rectum: 24.3 Small bowel: 6.1
Hebuterne 2014 [60]	Prospective	France	1903	Mean 59.3 (13.2)	Hospital	BMI	<75 years old: <18.5 ≥75 years old: <21	Pancreas: 66.7 Gastroesophageal: 60.2 Head and Neck: 48.9 Haematology: 34 Respiratory: 45.3 Ovaries/uterus: 44.8 Colorectal: 39.3 Breast: 20.5 Prostate: 13.9 Other disease sites: 30.0
Planas 2016 [43]	Cross-sectional	Spain	401	Mean 64.6 (14)	Hospital	Nutritional Risk Screening (NRS) 2002	NRS ≥ 3	Gastroesophageal: 47.4 Pancreas, liver and bile: 45 Respiratory: 42.9 Colorectal: 39.1 Hematology: 36.8
Muscaritoli 2017 [61]	Prospective	Italy	1951	Mean 62.7 (12.9)	Outpatient	Mini Nutritional Assessment (MNA)	<17	Gastroesophageal: 40.2 Pancreas: 33.7 Head and Neck: 23.8 Respiratory: 20.9 Genitourinary: 15.8 Unknown primary: 14.3 Colorectal: 13.4 Other GI: 13.2 Liver and bile ducts: 6.9 Breast: 5.8 Other cancers: 5.1
Li 2018 [62]	Cross-sectional	China	1138	Mean 60.6 (14.5)	Hospital	Nutritional Risk Index (NRI)	WL > 5% in 6 months or body mass index (BMI) < 20 kg/m^2^ with WL > 2%	Head and Neck: 67 Pancreas: 63 Gastroesophageal: 59.3 Colorectal: 45.1 Other disease sites: 36.3 Haematology: 36 Uterus/ovaries: 34.2 Kidney/bladder: 33.3 Respiratory: 32.1 Hepatobiliary: 31.6 Prostate/testicles: 28.6 Breast: 19
Na 2018 [63]	Prospective		1588		Hospital	Patient-Generated Subjective Global Assessment (PG-SGA)	B (moderately malnourished) C (severely malnourished)	Esophagus: 52.9 Pancreas and bile ducts: 47.6 Lung: 42.8 Stomach: 29.1 Liver: 24.7 Colon: 15.9
Marshall 2019 [64]	Prospective	Australia	1677	Two cohorts: 2012: mean 62.8 (13.5) 2014: mean 62.5 (13.8)	Hospital or oupatients	Malnutrition Screening Tool (MST) PG -SGA	MST ≥ 2 (risk of malnutrition) PG-SGA B or C	Breast: 19.6 and 21.5 * Colorectal: 18.6 and 15.2 * Haematological: 14.5 and 17.9 * Genitourinary: 10.2 and 8.1 * Upper gastrointestinal: 8.5 and 9.8 * Lung: 8.4 and 9.8 * Head and Neck: 6.5 and 6.1 * Skin and melanoma: 5.1 and 3.4 * Other: 4.5 and 4.3 * Gynaecological: 3.9 and 3.9 *
Álvaro Sanz 2019 [42]	Prospective	Spain	295	Median 62 (17)	Outpatient	Nutriscore	≥5 (at nutritional risk)	Gastroesophageal: 75 Pancreas-bile ducts: 70.6 Head-Neck: 33.3 Other 30.8 Gynecology: 28.6 Lung 26.6 Colorectal: 7.5 Breast: 0 Urotelial: 0

* 2012 and 2014 surveys.

By using the Nutritional Risk Screening (NRS 2002), Bozzetti et al. [59] and Planas et al. [43] reported an overall prevalence of increased nutritional risk up to 62.5% for esophagus and 66.7% for pancreatic cancers. Even higher rates (75% for gastroesophageal and 70.6% for pancreatic tumors) were demonstrated using a different risk screening tool [42]. A slightly lower prevalence has been shown applying scoring tools that allow to directly diagnose malnutrition. By using a combination of criteria based on BMI and percentage weight loss over time, Pressoir et al. [58] found an overall prevalence of malnutrition of 49.5% for upper digestive tumors. This finding was confirmed by an Italian study conducted in 2017 involving 1951 patients that used the mini nutritional assessment [2] and demonstrated a prevalence of malnutrition of 40.2%. In the same study, malnutrition was diagnosed in 33.7% of pancreas cancers. When using patient-generated global assessment (PG-SGA), the rates of malnutrition were generally concordant [63]. Only one study that also used PG-SGA showed significantly lower rates of malnutrition in all explored tumor types [64]. This finding may be attributable to the characteristics of the study cohort, which included mostly overweight patients, with non-metastatic disease and where potentially cachexia-inducing tumor types were under-represented (i.e., respiratory and upper gastrointestinal).

High prevalence of increased nutritional risk or overt malnutrition apply also to head and neck cancers. In this group, rates of increased nutritional risk ranging from 28.6% [59] to 67% [62] and of overt malnutrition in the range of 23.8–48.9% have been demonstrated [2,58,60].

When addressing lung tumors, increased nutritional risk has been reported in 26.6–42.9% of patients [42,43,59,62]. The higher risk of malnutrition associated with lung cancer is in agreement with the elevated prevalence of overt malnutrition shown by other studies [60,63,64] in the range of 20.9–45.3%.

Malnutrition is also frequently associated with hematologic malignancies with rates of 34–36.8% [58,60,63]. This is especially important from a prognostic and therapeutic perspective as malnutrition may worsen disease-related and treatment outcomes [65,66]. Mechanisms underlying malnutrition and wasting in this type of cancer are currently poorly understood. Among genitourinary tumors increased nutritional risk/malnutrition has been reported in up to 28.6% of patients with prostate/testicle neoplasms, up to 33.3% with kidney/bladder cancers, and up to 44.8% with bladder/uterus tumors [43]. Prevalence is generally lower in patients with colorectal and breast cancers [2,42,58,59,62,64], with some exceptions [43,60].

The prevalence of severe malnutrition, i.e., cachexia in cancer patients, has been reported by numerous studies [2,67,68]. Diagnosis of cancer cachexia is based on the detection of (a) unintentional weight loss >5% in the previous six months, or (b) a BMI < 20 kg/m^2^ associated with progressive weight loss (>2% in six months), or (c) a weight loss >2% in 6 months combined with low muscle mass [7]. Using these criteria, Muscaritoli et al. [2] found that the percentage of patients presenting with cachexia was much higher than that of those classified as malnourished by the mini-nutritional assessment, up to 70% in pancreatic and gastroesophageal cancers. A lower overall prevalence of 36% was reported by Blauwhoff-Buskermolen et al. [67] in a cohort of 241 patients with advanced mixed tumors, although the type of muscle measurement may have influenced the results. These data are in line with those reported by a recent systematic review including 21 studies [68] that showed a prevalence of cachexia in patients at risk for its development of 30% both in the U.S. and in Europe. The highest rates were demonstrated in the liver (50%), pancreas (45.6%) and head and neck cancers (42.3%).

Besides isolated unintentional weight loss or associated with loss of body fat, the spectrum of nutritional abnormalities in cancer patients also includes sarcopenia, defined by a reduction of muscle mass and function typical of the aging process [69]. Sarcopenia can be detected in cancer patients presenting with low, normal or increased BMI and has severe consequences on surgical complications, chemotherapy-induced toxicity and survival. A recent systematic review reported a prevalence of 38.6% of pre-therapeutic sarcopenia in a cohort of 6894 patients, with the highest rates in esophageal and lung tumors [14]. In locally advanced esophageal cancer, its prevalence ranges from 16% at diagnosis to 31% after adjuvant therapy and to 35% in survivors one year after diagnosis [70]. A slightly higher (44.6%) prevalence has been shown in older patients. Correlations with worse surgical outcomes and poor survival have been reported [12,15]. In lung cancer, its prevalence reaches 52.8% and it is associated with a lower overall response rate to chemotherapy and poorer progression-free survival [71]. The combination of both low muscle strength and mass affects 48.2% of older patients with head and neck cancer and it appears to be a better predictor of overall survival than the single criteria [72]. Similar observations have been reported for gastrointestinal cancers undergoing surgery, despite the heterogeneity in the assessment methods and criteria for sarcopenia diagnosis [73].

Overall, these data confirm the high risk and prevalence of malnutrition and cachexia in some cancer types, for which therefore special attention should be paid in the early disease stage. Due to the prognostic implications of malnutrition and low muscle mass on treatment tolerance, quality of life, and survival, routine screening and assessment of malnutrition should be warranted in all cancer patients, but especially in those affected by tumors localized in gastrointestinal pancreatic, head and neck, and lung districts.

## 4. The Impact of Treatment Phase and Treatment Type

### 4.1. Curative Setting: Surgery

Surgery is the mainstay of treatment in many solid tumors with more than 80% of patients requiring an operation in the treatment pathway [74]. In the curative setting, that is the clinical scenario in which cure can be obtained by surgical intervention, the occurrence of complications can dramatically influence the clinical outcome and also local control, with a higher risk of recurrence. For instance, anastomotic leak after rectal resection for cancer is strictly related to poorer survival. The prevention of complications is therefore crucial, and a central role is played by the evaluation and treatment of malnutrition, especially in patients undergoing major cancer surgery.

Malnutrition is indeed a modifiable risk factor for surgery. Perioperative nutritional support is very effective in decreasing non-infectious and infectious complications and also the length of hospital stay [75]. As already pointed out in this paper, impaired intake is the most important etiological factor in the development of malnutrition. Active intervention in the presurgical phase can impact adequate intake and treatment-related symptoms such as loss of appetite. Immunonutrition is gaining space within the pre-surgical phase, being it able to contribute to a reduction of surgical complications [6,76]. In cases of malnutrition deriving from tumor-related mechanical obstruction or malabsorption due to previous surgery, parenteral nutrition is to be considered to prepare the patient for surgery [77,78]. Moreover, sarcopenia, often associated with multimodal therapy, is definitively affecting pre-operative outcomes with an increased risk of postoperative complications in esophageal cancer [12,70,79].

Different cancer types are related to variable malnutrition status and also the definition of malnutrition can differ in the different surgical settings [80,81]. Tailored treatment, but also personalized evaluation according to cancer type is an intriguing concept. McKenna et al. [82] found that commonly available data, such as BMI and percent weight loss, could be used to risk-stratify patients undergoing major operations for different types of cancer. Unlike albumin or other data that are often missing, these parameters can always be obtained.

One upcoming issue is the variability amongst different cancer types regarding the most common specific definition of malnutrition. With all the limitations of a single study, they support the idea that, according to the cancer type, the malnutrition definition that best predicted postoperative risk differed for six cancer types and is: severe malnutrition for colorectal cancer, ESPEN 2 for esophageal cancer, ESPEN 1 for gastric, lung, and pancreatic tumors, and NSQIP for liver cancer. Accurate risk stratification for the type of cancer and type of surgery would therefore allow for rehabilitation in high-risk patients and perhaps improve outcomes. This introduces the concept of personalization, not only of treatment but also of evaluation according to cancer type. Unfortunately, the actual attention paid to nutritional status is far from the standard required in oncology treatment [83].

### 4.2. Curative Setting: Radiotherapy

Radiation treatment is employed as a curative treatment both in the postoperative setting and as an exclusive approach in many cancers. In recent years, the refinement of treatment schedules, technical improvements, and better association with systemic drugs have increased the potential of this therapeutic approach.

The balance between efficacy and toxicity has been one of the most discussed issues. Sometimes, the boundary between them is not so clearly defined and the therapeutic window may be narrow. In this regard, treatment of head and neck cancer patients represents one of the most challenging ones, as radiation therapy increases locoregional control and improves outcome, is also employed as an organ-preservation strategy, but it is burdened by many toxicities, mainly on the mucosal tract. This implies that weight loss is often a treatment-limiting toxicity if accurate nutritional programs are not fully implemented. Dysphagia, dysgeusia, pain swallowing, and mucositis represent the most frequent adverse events by radiation, amplified by concurrent systemic treatments. In a recent study, at population-based data, and considering the ESPEN-recommended weight loss grade, at the time of diagnosis grade 1–2 malnutrition was present in 33% of the patients, whilst grade 3–4 malnutrition was found in 24% of the patients [3]. Baseline nutritional status was shown to be an important determinant of reduced treatment intensity, lower quality of life, and worse outcomes in head and neck cancer patients [84,85]. Moreover, weight loss during treatment represents a major issue in head and neck oncology [60], increasing the rate of adverse events, often within a cluster of symptoms and signs, and hospitalizations. It is reported that more than 70% of head and neck cancer patients experience any grade of malnutrition during radiation [60]. Therefore, baseline weight loss grade, type of radiation treatment in terms of dose and fields, the addition of concurrent systemic treatment, swallowing ability, and presence of caregivers should all be factors to consider before the commencement of radiation, to identify a tailored nutritional support program.

It should be underlined that similar consequences of malnutrition have been reported also in other cancer subsites, where weight loss at baseline and during radiation are common treatment-induced toxicities. In esophageal cancer, malnutrition is a frequent hallmark of patients at diagnosis, and nutritional support has been extensively suggested, particularly for patients undergoing radiation therapy [58,59]. Moreover, lung cancer [86] and pancreatic cancer [87] patients suffer from nutritional impairments when undergoing radiation therapy. Thus, nutritional programs are often suggested and have a rationale for these cancer types [88,89].

### 4.3. Oncological Systemic Treatments (Chemotherapy/Targeted Agents/Immunotherapy)

Malnutrition during oncological systemic treatments may expose patients to a dangerous, vicious circle. In fact, from one side, malnutrition is a consequence of the treatments themselves, as one of the possible side effects. Dysgeusia, nausea, vomiting, anorexia, mucositis, and dysphagia induced by chemotherapy are just some toxicities that may result in malnutrition. On the other side, the lack of an adequate nutritional status puts the patients at higher risk of developing toxicities, and it is one of the major causes of poor tolerance to the therapies [59]. Moreover, the association between nutritional status and response to treatments is well-known. This effect has been documented in different cancer settings [59].

In patients undergoing chemotherapy, the presence of gastroenteric symptoms is closely linked to unintentional weight loss [90]. Among different subsites, gastrointestinal cancers undergoing chemotherapy present the highest incidence of malnutrition, both due to the peculiarity of these diseases and to the administered chemotherapy, frequently composed of drugs causing mucositis and diarrhea [91,92]. Moreover, head and neck and lung cancer patients are among the most exposed to nutritional issues, due to the burden of disease and the consequent inflammatory status. In this regard, chemotherapy-induced toxicities often worsen the metabolic and nutritional status [6,93]. Theoretically, targeted treatments should preserve from nutritional issues, in that they should hit specific targets not involved in the absorption of nutrients. However, this principle is often disregarded, as many targeted agent-induced toxicities are represented by anorexia, mucositis, diarrhea, dysgeusia and dysphagia. Still, the exact prevalence of malnutrition induced by targeted treatment is not so precisely quantifiable, as we lack enough data about the direct effect of this class of drugs in causing nutritional problems. One exception is represented by hedgehog inhibitors, directly inhibiting taste receptors, thus causing nutritional troubles. As a matter of fact, dysgeusia and weight loss are reported in about half and one-third of the treated patients [94,95].

The exact prevalence of nutritional issues induced by immunotherapy has not been comprehensively studied. Some adverse effects caused by immune checkpoint inhibitors could justify a causal role of this treatment in reducing intake and absorption of nutrients: diarrhea, pancreatic insufficiency, nausea and fatigue might reduce the caloric intake and produce weight loss. On the other side, there remains evidence showing that cancer-induced chronic inflammation and impairment of nutritional intake impair the quality of immune response, both innate and adaptative [96]. It is therefore expected that this field of research will substantially increase in the near future.

### 4.4. End-of-Life Period

There is no consensus on the definition of end-of-life. Indeed, the term may refer to “people at risk of dying within the next 12 months” according to the guidance of the General Medical Council of 2010 (4) or to “the final weeks, days, hours of a patient’s life” according to the NHS document on end-of-life care strategy [97].

It is therefore clear that life expectancy is a very important parameter, but it is often very difficult to establish it.

Cancer patients at the end of life are often characterized by a worsening of nutritional status determined by a gradual reduction in food intake and progressive weight loss. The causes may be multifactorial, directly linked to the neoplastic disease or its treatment, such as anorexia, nausea, vomiting, dysgeusia, dysphagia, diarrhea, or linked to obstruction of the gastroenteric tract. Many patients develop severe malnutrition and dehydration [98,99].

Regarding end-of-life as a life expectancy of a few weeks, every intervention should be considered together with ethical aspects. Such intervention should be non-invasive and limited to symptom management considering personal and family expectations and the benefits of quality of life [6].

## 5. Conclusions and Recommendations

The spectrum and magnitude of nutritional disorders in oncological patients vary according to cancer type, disease setting, comorbidities, and type of treatment performed. The prevalence of weight loss and associated symptoms is quite heterogeneous and needs to be assessed with recognized criteria, thus allowing a clear classification and standardization of therapeutic interventions. The analysis of the literature has not been conducted with the criteria of a systematic review, as the purpose of this paper is to shed light on a complex issue with the eyes of different expertise.

Therefore, we state that since nutritional issues have a relevant impact on patient quality of life, treatment intensity, and disease outcome, it is important that both oncologists and nutritionists share and address the following key points:

− Malnutrition is highly prevalent in certain cancer types and at advanced stages. Early screening and periodical reassessment are mandatory.
− Personalized choice of best malnutrition definition according to cancer site allows an accurate risk stratification for postoperative complications and targeted preoperative interventions.
− In radiotherapy, the boundary between efficacy and toxicity is not so clearly defined. Therefore, careful monitoring and implementation of nutritional support prevent weight loss-associated adverse complications.
− Nutrition-related problems are common during chemotherapy, but their exact prevalence during targeted treatments is generally unknown. This does not hold true for selected agents, such as hedgehog inhibitors, which extensively affect nutritional intake and status by specific mechanisms. Appropriate interventions should be provided in a timely and appropriate manner.
− End-of-life period interventions should be targeted at patient comfort and quality of life.

## Figures and Tables

**Table 1:** Phenotypic Criteria for malnutrition diagnosis.

Phenotypic Criteria		
Weight Loss (%)	Low Body Mass Index (kg/m^2^)	Reduced Muscle Mass
>5% within past 6 months Or >10% beyond 6 months	<20 if <70 years, or <22 if >70 years Asia: <18.5 if <70 years, or <20 if >70 years	Reduced by validated body composition measuring techniques

**Table 2:** Etiologic Criteria for malnutrition diagnosis.

Etiologic Criteria	
Reduced Food Intake or Assimilation	Inflammation
≤50 % of ER > 1 week, or any reduction for >2 weeks, or any chronic GI condition that adversely impacts food assimilation or absorption	Acute disease/injury or chronic disease-related

**Table 3:** Criteria for the severity of malnutrition staging.

	Phenotypic Criteria		
	Weight Loss (%)	Low Body Mass Index (kg/m^2^)	Reduced Muscle Mass
Stage 1 / Moderate Malnutrition (Requires 1 phenotypic criterion that meets this grade)	5–10% within past 6 months Or 10–20% beyond 6 months	<20 if <70 years, or <22 if >70 years	Mild to moderate deficit
Stage 2 / Severe Malnutrition (Requires 1 phenotypic criterion that meets this grade)	>10% within past 6 months Or >20% beyond 6 months	<18.5 if <70 years, or <20 if >70 years	Severe deficit

## Data Availability

Not applicable.
